# Mating behaviors in ovoviviparous black rockfish (*Sebastes schlegelii*): molecular function of prostaglandin E2 as both a hormone and pheromone

**DOI:** 10.1007/s42995-023-00214-w

**Published:** 2024-01-19

**Authors:** Likang Lyu, Yijia Yao, Songyang Xie, Xiaojie Wang, Haishen Wen, Yun Li, Jianshuang Li, Chenpeng Zuo, Shaojing Yan, Jingyi Dong, Xin Qi

**Affiliations:** 1 Key Laboratory of Mariculture (Ocean University of China), Ministry of Education, Ocean University of China, Qingdao, 266003 China; 2 Institute of Evolution and Marine Biodiversity, Ocean University of China, Qingdao, 266003 China

**Keywords:** Black rockfish, Mating behavior, Ovoviviparity, Prostaglandin E2

## Abstract

Prostaglandins (PGs) are profound hormones in teleost sexual behavior, especially in mating. PGs act as pheromones that affect the olfactory sensory neurons of males, inducing the initiation of a series of mating behaviors. However, the molecular mechanism by which PGs trigger mating behavior in ovoviviparous teleosts is still unclear. In the present study, we employed the ovoviviparous black rockfish (*Sebastes schlegelii*), an economically important marine species whose reproductive production is limited by incomplete fertilization, as a model species. The results showed that when the dose of PGE_2_ was higher than 10 nmol/L, a significant (*P* < 0.05) increase in mating behaviors was observed. Dual-fluorescence in situ hybridization indicated that PGE_2_ could fire specific neurons in different brain regions and receptor cells in the olfactory sac. After combining with specific neurons in the central nervous system (CNS), a series of genes related to reproduction are activated. The intracerebroventricular administration of PGE_2_ significantly increased *lhb* levels (*P* < 0.05) in both sexes. Moreover, steroidogenesis in gonads was also affected, inducing an increase (*P* < 0.05) in E_2_ levels in males and T levels in females. PGE_2_ levels were also increased significantly (*P* < 0.05) in both sexes. The present study revealed that PGE_2_ can activate mating behavior in black rockfish in both hormone and pheromone pathways, leading to variations in sex steroid levels and activation of reproductive behaviors. Our results provide not only novel insight into the onset of mating behaviors in ovoviviparous teleosts but also solutions for the incomplete fertilization caused by natural mating in cage aquaculture.

**Supplementary Information:**

The online version contains supplementary material available at 10.1007/s42995-023-00214-w.

## Introduction

Sexual behavior is one of the most profound activities in sexually reproducing animals. Both sexes process special behavior patterns to release mature gametes for fertilization, leading to the creation of offspring. In teleosts, a series of behaviors are performed during the process of gamete release. These behaviors are named differentially depending on the pattern, such as courtship, chasing, contact, spawning, sperm release, and oviposition. They are described as “sex behavior” or “reproductive behavior”, meaning a series of behavioral acts that are performed by sexually mature females and males ultimately for the production of offspring (Munakata and Kobayashi 2010). Because the sexual behaviors of teleosts are much more derived from nature than learned, they can be performed without experience after sexual maturation. Researchers have speculated that fish might use chemicals including hormones (Munakata and Kobayashi 2010) or odorous molecules (Chung-Davidson et al. 2011), etc. to process sexual behaviors (Stacey and Sorensen 2011).

After gamete maturation, female fish usually ovulate and spawn. Meanwhile, mature males are attracted by females to start a series of mating behaviors, leading to fertilization (Kobayashi et al. 2002; Stacey et al. 2003). Hormones and neuropeptides usually play a crucial role in these processes. In general, these hormones and neuropeptides are classified into three categories. The first one is regarded as a “potentiator” (Munakata and Kobayashi 2010). A potentiating hormone has no direct effect on behavior and is not essential for the occurrence of behavior. Gonadotropin-releasing hormone (GnRH) is a typical potentiator hormone that can enhance subsequent spawning but is dispensable for sexual behavior (Volkoff and Peter 1999). The second category is characterized as a “requirement” or “primer” (Munakata and Kobayashi 2010). As the name indicates, this hormone does not activate sexual behavior, but is essential for the occurrence of the behavior. Sex steroid hormones, especially estrogen and androgens, basically function as priming agents. Studies on zebrafish (*Danio rerio*) have shown a correlation between high levels of both 11-ketotestosterone (11-KT) and 17-β estradiol (E_2_) and mating behaviors (Pradhan and Olsson 2015). In addition, when androgen is injected as a priming agent, male behavior can be triggered by other external cues (Yambe et al. 2003). The third and most important trigger is named the “physiological trigger”, which activates sexual behavior directly and rapidly when physiological and environmental conditions are appropriate (Munakata and Kobayashi 2010). One dominant trigger hormone is prostaglandins (PGs). In 1988, PGF_2α_ and its metabolite 15-keto-PGF_2α_ were first reported as being released from ovulated female goldfish (*Carassius auratus*) to initiate male courtship by activating olfactory sensory neurons (OSNs) (Sorensen et al. 1988). In oviparous medaka (*Oryzias latipes*) and goldfish, ovary-produced PGs can act on follicular cells to promote ovulation. Meanwhile, excess PGs are released into the environment to attract males (Appelt 1995; Fujimori et al. 2012). A study on the cichlid fish *Astatotilapia burtoni* showed that PGF_2α_ injection can activate a naturalistic pattern of sexual behavior in females, which transduces signals to cells in the brain (Juntti et al. 2016). In addition, PGF_2α_ can also act as a pheromone to trigger zebrafish reproductive behavior via the olfactory system (Yabuki et al. 2016). It is suggested that PGs play a key role in teleost mating. Apart from serving as pheromones released from females to attract males, there is also strong, robust evidence that PGs also play important roles as hormones in ovulation and spawning in teleosts (Criscuolo-Urbinati et al. 2012; Stacey et al. 2003; Takahashi et al. 2018). PG signals from the reproductive tract can communicate with the brain (Juntti et al. 2016; Saper et al. 2012), especially in the preoptic area (POA), which is thought to be related to sexual behavior across vertebrates (Goodson 2005; Wong et al. 2012). Ovariectomized peacock blenny (*Salaria pavo*) shows a reduction in the expression of sexual behaviors toward males, but administering PGF_2α_ resulted in recovery of the frequency of sexual behaviors (Gonçalves et al. 2014). In goldfish, injection with PGF_2α_ in males and females can induce typical female spawning behavior (Kobayashi et al. 2002; Saoshiro et al. 2013).

In oviparous teleosts, the mating process is always associated with female ovulation activated by PGs, and the gametes are expelled into the water environment for in vitro fertilization. However, in ovoviviparous teleosts, the gametes are always asynchronously mature (Stacey and Sorensen 2011). Early studies on ovoviviparous teleost guppies (*Poecilia reticulata*) showed that exposure of adult males to E_2_ or xenoestrogen (4-*tert*-octylphenol) could cause a significant decrease in the intensity and rate of sexual display (Bayley et al. 1999), and that estrogen can restore the sexual receptivity of ovariectomized females (Liley 1972). Administration of an aromatase inhibitor, fadrozole, was shown to reduce male sexual behavior in guppies (Hallgren et al. 2006). Black rockfish (*Sebastes schlegelii*), an economically important marine species, is an ovoviviparous teleost with long-term sperm storage. As an ovoviviparous teleost, gamete maturation in black rockfish is asynchronous. Spermatogenesis usually commences in late July and lasts until December, when mating occurs. After the mating process by the modified urogenital papillae of males, sperm are stored in the ovary cavity when oocytes are still undergoing the vitellogenesis period (Mori et al. 2003; Wang et al. 2021). When the oocytes are finally mature in late March, stored sperm activate and fuse with the oocytes. Females usually undergo an approximately 25-day pregnancy period (depending on the water temperature), and fertilized eggs develop into larvae in the ovary before final parturition (Lyu et al. 2022). In contrast to oviparity, this special reproductive strategy renders artificial reproduction difficult in black rockfish. As gamete maturation is asynchronous, fertilization is dependent on natural mating, which limits artificial insemination and the optimization of black rockfish. Artificial insemination of black rockfish invariably results in incomplete fertilization. A previous study showed that this incomplete fertilization was caused by a lack of sufficient sperm storage, and that the amount of sperm amount was related to the frequency of mating (Yao et al. 2023). Normally, a mother black rockfish could have over 50,000 fries at the same time, which means over 50,000 mature oocytes were ready for fertilization. However, if the frequency of mating is lower than normal, a proportion of the mature oocytes will not be fertilized. Mature sperm of internally fertilized black rockfish were observed swimming in female ovary fluid after mating and then stored in the crypts outside the follicular layer (Liu et al. 2019; Zhao et al. 2021). However, the female gamete is still mature during the mating season, which makes the mating initiation mechanism different from that of oviparous teleosts. There is still a lack of literature explaining the mechanism of mating initiation. In a previous study on black rockfish, COX1-2, which is a PG biosynthesis-limited enzyme, was significantly upregulated during the vitellogenesis stage when mating started, implying a potential role in mating behavior (Lyu et al. 2022). Nevertheless, a comprehensive understanding of the PG mechanism in black rockfish mating behavior is still lacking. For example, PGs function as pheromones to affect behavior alternation or as endogenic hormones that affect the hypothalamus–pituitary–gonadal axis (HPG axis) and sex steroid hormones. Prior to the present study, we tested the function of different PGs (PGF_2α_, PGE_2_ and PGD_2_) and steroid hormones (E_2_ and T) as pheromones in triggering a series of mating behaviors in black rockfish. Of these, only PGE_2_ altered the behavioral pattern. In the present study, we investigated the role of PGE_2_ in triggering mating in black rockfish. The molecular mechanism of PGE_2_ in the brain and gonads was further analyzed. The present study is the first to identify PGE_2_ as the functional pheromone for triggering mating behavior in ovoviviparous black rockfish, and mechanism by which it acts at the molecular level. Our research will provide novel information for increasing our understanding of reproduction in ovoviviparous teleosts and provide a theoretical basis for artificial reproduction in ovoviviparous teleosts.

## Materials and methods

### Animal collection and treatment

Black rockfish (1200 ± 300 g) were obtained from marine cages in the northern Yellow Sea, Shandong Province, China. All procedures involved in the experimental treatment of individuals were approved by the Animal Research and Ethics Committees of Ocean University of China before the initiation of the study. Animal experiments were performed in accordance to the relevant guidelines. The experimental individuals described below were anesthetized with ethyl 3-aminobenzoate methanesulfonic acid (MS-222, 200 mg/L) before sacrifice.

### Observation of patterns of behavior and assay of black rockfish after PGE_2_ bath

Three pairs of adult male and female black rockfish were obtained in September 2020 during the mating season. One pair of fish individuals was placed into a glass tank (1 m × 1 m × 1 m, 600 L water volume, 14 ± 1 °C, photoperiod 14 L:10 D, water salinity 28) with three surveillance cameras, one each on the X axis, Y axis and Z axis. (After two days, different concentrations of PGE_2_ were added to the tank at 21:00. In detail, PGE_2_ dry powder (Shanghai Yuanye Bio-Technology, China) was dissolved in ethanol as a stock solution (10^8^ nmol/L). A 0.6 mL aliquot of either pure ethanol (control) or of a working solution of PGE_2_ (10^7^ nmol/L, or 10^8^ nmol/L) was added to the tanks to give final concentrations of PGE_2_ in 0, 10 nmol/L and 100 nmol/L, respectively. Changes in behavior of individuals were observed and recorded using the surveillance cameras. These data were used to create heatmaps of the behavioral patterns at 15 min intervals.

### Intracerebroventricular (ICV) administration of PGE_2_

Nine female and nine male black rockfish were obtained in September 2020, i.e., during the mating season, for ICV administration of PGE_2_. The skull of each individual was trepanned with a 1 mm^2^ hole approximately 1 cm above the midpoint of the two eyes. The individuals were then divided into three groups, each comprising three males and three females. 10 mg of dry PGE_2_ powder (Shanghai Yuanye Bio-Technology, China) was dissolved in 1 mL of ethanol. The PGE_2_ stock solution (10 μg/μL) was then diluted with phosphate buffered saline (PBS) and administered by ICV at concentrations of 0, 0.01 ng/g, and 0.1 ng/g wet body weight by injection through the hole. The injected PBS had the same volume of ethanol in each group. After injection, the hole was filled with dental plaster to prevent water seepage. Tissues and blood samples were collected after 6 h ICV. The sample collection protocol was as described in Sect. "Animal collection and treatment".

### DNA isolation, microsatellite primers screening and PCR

Thirty pregnant individuals were sacrificed for pregnancy rate measurement. Briefly, the whole ovary was weighed before mixing the embryo within. The mixture was then sampled randomly to weigh the absolute brood amount and estimate the pregnancy rate. This process was replicated four to six times. To test the polymorphism of six microsatellite primers (KSs7, Ssc12, Ssc23, Ssc51, Ssc69, and Sra7-7), the genomic data of 230 embryos and 10 females were analyzed. DNA isolation was performed by a TIANamp Marine Animals DNA Kit (TIANGEN, China, Beijing) according to the manufacturer’s instructions.

Microsatellite primers were selected from previously published studies (An et al. 2009; Gao et al. 2018), and the 5′ end of each forward primer was labeled by ROX, FAM and HEX (Table 1). The number of alleles (Na), polymorphism information content (PIC), expected and observed heterozygosity (*He*, *Ho*), and Hardy–Weinberg equilibrium (HWE) were calculated by Cervus 3.0 (Table 1).

**Table 1:** Characterization and genetic diversity parameters of microsatellite loci

Locus	Accession no.	Repeat motif	Size range (bp)	Ta (℃)	Na	Ho	He	PIC	HW	Primer sequences
KSs7	EF109806 (GenBank)	(GT)_16_	174–230	54	16	0.750	0.887	0.874	**	F: ROX-TGGGCAATAAATAAGAGAGGA
										R: CCGTCTGCAATCTGACTCA
Ssc12	AB058405 (DDBJ)	(AC)_20_	152–238	59	14	0.804	0.816	0.798	NS	F: FAM-AACACGCTGAACAGAGAACAAA
										R: GCTCCGACTATAGCTGGTCCTA
Ssc23	AB058406 (DDBJ)	(TG)_21_	177–279	57	16	0.946	0.914	0.904	ND	F: HEX-AGTGTCATGCCCTCTTCCAG
										R: CACTCGGCATTCTCACCTCA
Ssc51	AB058407 (DDBJ)	(GT)_20_T(TG)_5_	168–264	57	15	0.902	0.870	0.853	*	F: ROX-GTGCTGATGGAAAACACTACCA
										R: GTGACCTTTCCCTGAACACACT
Ssc69	AB058408 (DDBJ)	(GT)_13_	138–160	57	9	0.929	0.829	0.804	***	F: HEX-GGCACCGAGCTCAACCTTACTG
										R: TGCTGTGACTATTTCCCTCTGGC
Sra7-7	AF269055 (GenBank)	(CA)_12_	195–220	57	6	0.571	0.617	0.574	NS	F: FAM-GCATGAAAGTGTATGAAAGGC
										R: CATGTGATTCTGTGTCTAACTGAG

Fluorescent dye labels: FAM (blue), ROX (red), and HEX (green)

*NS* represents no statistically significant difference (*P* > 0.01), *ND* represents non-detectable

* represents statistic difference (*P* < 0.05)

** represents statistically significant difference (*P* < 0.01)

*** represents highly statistically significant difference (*P* < 0.001)

### RNA isolation, reverse transcription, and qPCR

Ovary (O), brain region (Telencephalon, TC; Diencephalon, DC; Valvula cerebelli, VCe; Pituitary, P; Corpus cerebelli, CCe; Pons, Po; Medulla oblongata, MO) Testis (T), Urogenital papillae (UP), and olfactory sac (OS) samples from 24 individuals, including three males and three females for *ptger* distribution and nine males and nine females for ICV, were placed in 1 mL TRIzol solution (Vazyme, Nanjing, China) with solid-glass beads and homogenized by a high-throughput tissue lyser (DHSbio, Beijing, China). Total RNA was extracted according to the TRIzol manufacturer’s instructions (Vazyme, Nanjing, China). Qualities and concentrations of total RNA were measured by agarose gel electrophoresis and a biophotometer (OSTC, Beijing, China). Total RNA was reverse transcribed into complementary DNA (cDNA) via the HiScript III 1st Strand cDNA Synthesis Kit (Vazyme, Nanjing, China) according to the manufacturer's instructions.

qPCR was performed on a StepOnePlus Real-Time PCR System (Thermo Fisher Scientific, USA) using ChamQ SYBR Color qPCR Master Mix (Vazyme, Nanjing, China) according to the manufacturer's instructions. All primers used in the present study are listed in Table 2. After initial denaturation at 95 ℃ for 30 s, each template was amplified with 40 cycles of denaturation for 5 s at 95 ℃ and annealing for 30 s at 60 ℃. The expression level of the target gene was calculated with the 2^−ΔΔct^ method. The expression levels were normalized against the 18S rRNA.

**Table 2:** Primer sequences used for ORF cloning, DISH, and qPCR

Primers	Sequence (5′–3′)	Tm (℃)	Products length (bp)	Accession no.	Amplification efficiency (%)
Primer for qPCR					
ptger EP1-F	GATGGAGGGCACCGAAAC	58	461	OP485622	105.6
ptger EP1-R	AGCGAACAGAGCGGAACG	59.4			
ptger EP2-F	TGAACGCTGGCGAAACGG	63.5	172	OP485623	110.3
ptger EP2-R	CTGTTGGACCTCGCCTTC	55.7			
ptger EP3-F	CGCTGCTGCCCGTCATAGGT	65.6	252	OP485624	110.0
ptger EP3-R	TCTCCGTGGTGAGCCGTTCC	65.3			
ptger EP4-F	TCTTCTCTGTGGCCGGGC	61.4	207	OP485625	94.1
ptger EP4-R	ACCAAGTGTCCGGGTATTGTT	58.5			
*sgnrh-F*	GTGTTGTTATTGGCGTTGGT	55.8	174	MN082617.1	93.93
*sgnrh-R*	AAGTCTCTCTTGGGTCTGGG	55.5			
*cgnrh-F*	TGCTGCTTGGGCTGCTTCTATGT	66.1	123	MN082616.1	91.5
*cgnrh-R*	CCTCTGAAACCTCTGATGTGCCG	65.7			
*kiss1*-F	ATCAGGAAATACTCAAAGCCC	55.5	201	KJ139960.1	92.00
*kiss1*-R	AGGAGTTGAGGTTGTATGAG	48.6			
*lhb*-F	TCCCCGTGATGTTGAGTTGG	61.1	140	AY609080.1	99.87
*lhb*-R	TGACACTTGGAACAGCCCTC	58			
*fshb*-F	AAGCTCTACAGGCATCTGCG	58.1	157	AY609079.1	105.23
*fshb*-R	TGAATTGGGTTTGGGTGCAG	61.4			
*fshr*-F	AGCAGGAACGAATCGAGGTG	60	181	JN165365.1	99.01
*fshr*-R	TGATCCAGATGAGGACCCGT	59.6			
*lhr-F*	GGAGCTGTCGGTCTACACAC	54.9	178	HQ712166.2	99.81
*lhr-R*	GCCAGAGGTGTCTCGATGTC	57.3			
*cox2*-F	CCAGGGAACAGATGATTACG	55.3	145	MT862758	100.03
*cox2*-R	CTTGAAGTGGGTGAGCAG	50.9			
*star-F*	CTGGCATCTCCTATCGGCA	59.1	179	MN082621.1	99.77
*star-R*	CTCCACACTATCTGTCCCA	50.1			
*cyp11a1-F*	AACAAATGGACCACGGACCTC	61	325	MW000347.1	95.94
*cyp11a1-R*	CTGGGTAGGTCTTTGGAGTGC	58.8			
*cyp19a1a-F*	GCACCGCCAGCAACTACTACA	61.3	325	FJ594995.2	99.39
*cyp19a1a-R*	GCCAAACTGTCCAGGTCGTCC	63.9			
*18s*-F	CCTGAGAAACGGCTACCACAT	59.3	119	KF430619.1	101.9
*18s*-R	CCAATTACAGGGCCTCGAAAG	61.4			
Primers for ISH prober preparation					
EP2-DISH-F	CGCATTTAGGTGACACTATAGAAGCGGCCACCATGTCGCTTCT	53.8	420	OP485623	
EP2-DISH-R	CCGTAATACGACTCACTATAGGGAGACATTGGACCTCGCCTTCAC	68			
*c-fos*-DISH-F	CGCATTTAGGTGACACTATAGAAGCGTCAACACGGAGTGCGATTC	57.1	700	PRJNA573572	
*c-fos*-DISH-R	CCGTAATACGACTCACTATAGGGAGACAGTTGGCTGGCTGGAAGTG	71			

### Colocalization of *c-fos* and *ep2* by dual-fluorescence in situ hybridization (DISH)

DISH for *ep2* and *c-fos* were performed to confirm the expression of EP2 on neuronal activity using previously described methods with modifications (Lyu et al. 2021). Briefly, the brain and olfactory sac were incubated in 0.1 mg/mL PGE_2_ (five males and five females) or solvent (five males and five females) for 5 min. Samples were then collected and fixed with buffered 4% paraformaldehyde (PFA) for approximately 24 h and embedded in paraffin. Subsequently, 7-mm thick sections were prepared for the DISH experiment. The probes for *ep2* and *c-fos* were labeled with digoxigenin (DIG)-dUTP and biotin-dUTP (Roche Diagnostics, Mannheim, Germany), respectively. After hybridization with DIG and biotin-labeled probes and post-hybridization steps, sections were blocked with 10% goat serum (Invitrogen, Carlsbad, USA). The blocked sections were incubated with a horseradish peroxidase (HRP)-conjugated anti-DIG antibody (diluted 1:500 in the blocking reagent) and rinsed twice with sterile PBS for 5 min each time. Chromogenic reactions were then performed using a tyramide kit with Alexa Fluor 488 (Invitrogen, Carlsbad, CA, USA) for approximately 30 min. The second fluorescence detection started after the first reaction appeared to produce appropriate results. The sections were incubated with 3% hydrogen peroxide for 1 h to inactivate conjugated HRP. Sections were then incubated with HRP-conjugated streptavidin (Proteintech, Chicago, USA). The final chromogenic reaction was performed using a tyramide kit with Alexa Fluor 594 (Invitrogen, Carlsbad, CA, USA) for approximately 30 min and stopped using a stop reagent (Invitrogen, Carlsbad, USA) to detect the signal. The sections were stained with DAPI for 10 s (10 mg/mL, Solarbio, Beijing, China) and then mounted in antifade mounting medium (Beyotime, Shanghai, China). Images were captured using an Olympus BX53F fluorescence microscope (Olympus, Japan). Digital images of DISA were processed by ImageJ 1.53 software (Wayne Rasband, National Institutes of Health, Bethesda, MD, USA) (Young and Morrison 2018).

### Hormone concentration measurement by radioimmunoassay and ELISA

To assess the PGE_2_, E_2_ and testosterone (T) levels after ICV treatment, blood samples from each individual were collected. PGE_2_ levels were measured by commercial ELISA kits (Runyu, Shanghai, China) according to the manufacturer’s instructions. RIA was performed to assay T and E_2_ levels by Iodine ^[125I]^ RIA kits (BNIBT, Beijing, China) according to the manufacturer’s instructions. The binding rate is highly specific with low cross-reactivity to other steroids, which was less than 0.1% for most circulating steroids.

### Statistical analysis

All data are expressed as the mean ± SEM. Data analyses were performed by nonparametric T test and one-way ANOVA followed by Dunnett T3 and the LSD multiple range test. Significant differences were considered at *P* < 0.05. The methods of statistical analyses were chosen according to previous reports (Björnsson et al. 2018; Davis et al. 2010; Du Toit et al. 2018). All statistical analyses were performed by SPSS 19.0 software (SPSS, Chicago, USA) and GraphPad Prism 9.3.1 (GraphPad Software, USA).

## Results

### Estimation of pregnancy rates in black rockfish

**Fig. 1:**
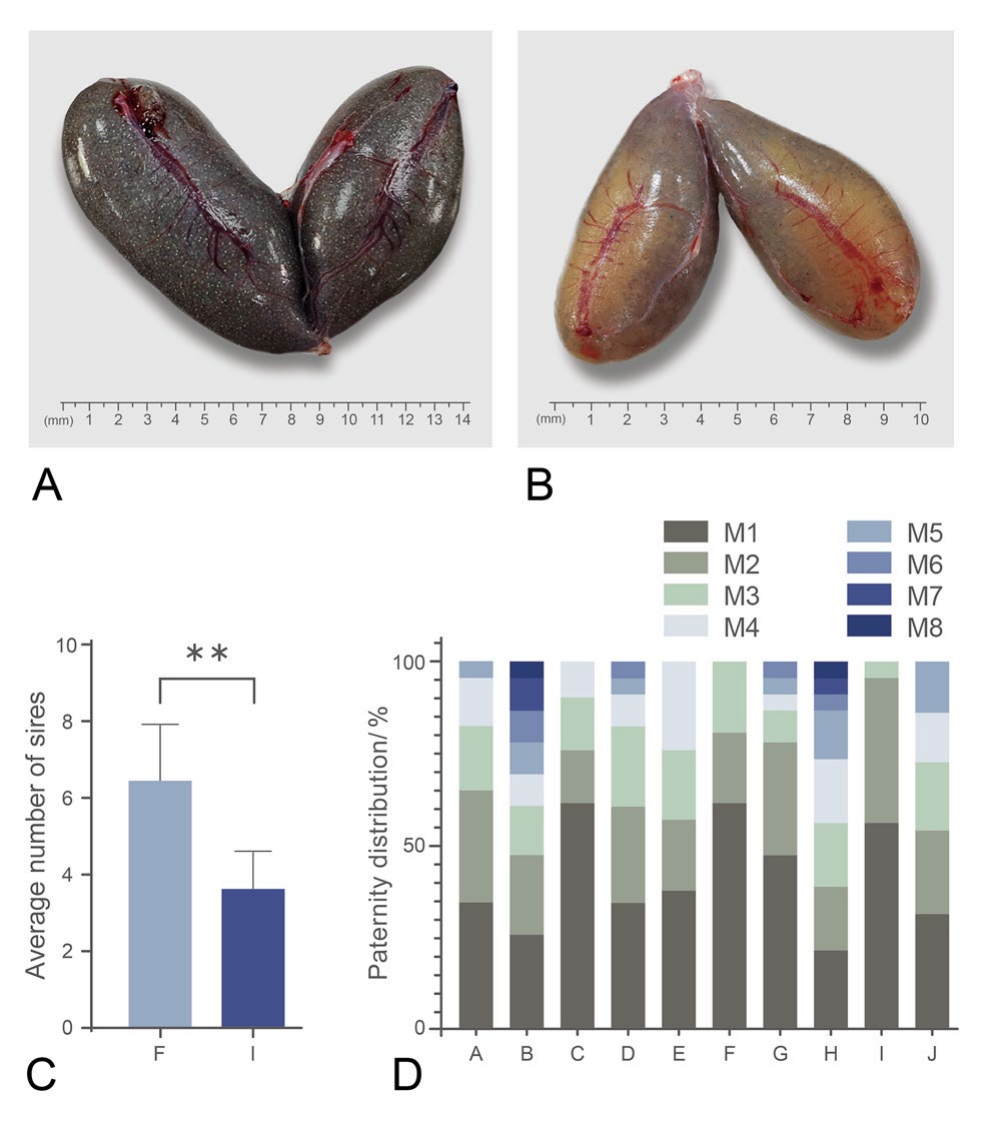
Multiple paternity analysis of black rockfish. **A** Dorsal ovary following complete fertilization. **B** Dorsal ovary following incomplete fertilization. **C** Comparison of paternity number in mothers with different fertilization situations. **Indicates significant difference between two groups (*P* < 0.01). **D** Proportions of paternity in different individuals. A–J represent individual mothers. M1–M8 represent the assumed male parent

Thirty pregnant individuals were randomly selected to measure the biological index and pregnancy rate. The average body weight was 1010.70 ± 202.64 g, body length was 36.40 ± 2.15 cm, and fatness was 2.07 ± 0.18. The average absolute brood amount was 97,720.32 ± 35,948.27, which was significantly positively correlated with average body weight (*r*^2^ = 0.7753, *P* < 0.05) and average body length (*r*^2^ = 0.5493, *P* < 0.05). Eleven out of 30 pregnant females examined were observed to have incomplete fertilization (36.67%). The lowest pregnancy rate of incompletely fertilized individuals was 37.81% (Supplementary Table 1). Figure 1A and B illustrates ovaries with complete or incomplete fertilization, respectively.

### Multiple paternity analysis

Genotypes at six microsatellite loci were analyzed from ten mothers and 223 embryos. As shown in Table 3, multiple paternity existed in both the incomplete and the complete fertilization groups (five mothers each). The number of sires in one female ranged from 3 to 8, with an average of 5.2 sires per brood. Furthermore, the sire numbers in the complete fertilization group were significantly higher than those in the incomplete fertilization group (*P* < 0.01. Figure 1C). The binomial skew index (B index) was employed to predict the existence of paternal advantage. The B index of seven out of ten broods was over 0, and four broods (C, F, G, I) were significantly skewed from equal paternal contributions (*P* < 0.05, Table 1). Figure 1D shows the paternity distribution of ten mother black rockfish.

**Table 3:** Paternity distribution and dominant paternal deviation index of female

Mother	Embryos	Number of sires	M1	M2	M3	M4	M5	M6	M7	M8	*B* value	*P*
A	23	5	8	7	4	3	1				0.028	0.1350
B	23	8	6	5	3	2	2	2	2	1	0.0014	0.4011
**C**	**21**	**4**	**13**	**3**	**3**	**2**					0.1474	0.0019
D	23	6	8	6	5	2	1	1			0.0447	0.0531
**E**	**21**	**4**	**8**	**4**	**4**	**5**					− 0.0113	0.5926
**F**	**21**	**3**	**13**	**4**	**4**						0.0907	0.0248
G	23	6	11	7	2	1	1	1			0.1317	0.0004
H	23	8	5	4	4	4	3	1	1	1	− 0.0024	0.5194
**I**	**23**	**3**	**13**	**9**	**1**						0.1122	0.0057
**J**	**22**	**5**	**7**	**5**	**4**	**3**	**3**				− 0.0132	0.6585

M1–M8 represent different mother individuals. C, E, F, I, and J were incomplete fertilization group. *B* value represents binomial deviation index, and *P* represents reliability. Under the condition of *B* > 0, smaller *P* values represents higher reliability of results

### PGE_2_ triggers a series of mating behaviors in black rockfish

**Fig. 2:**
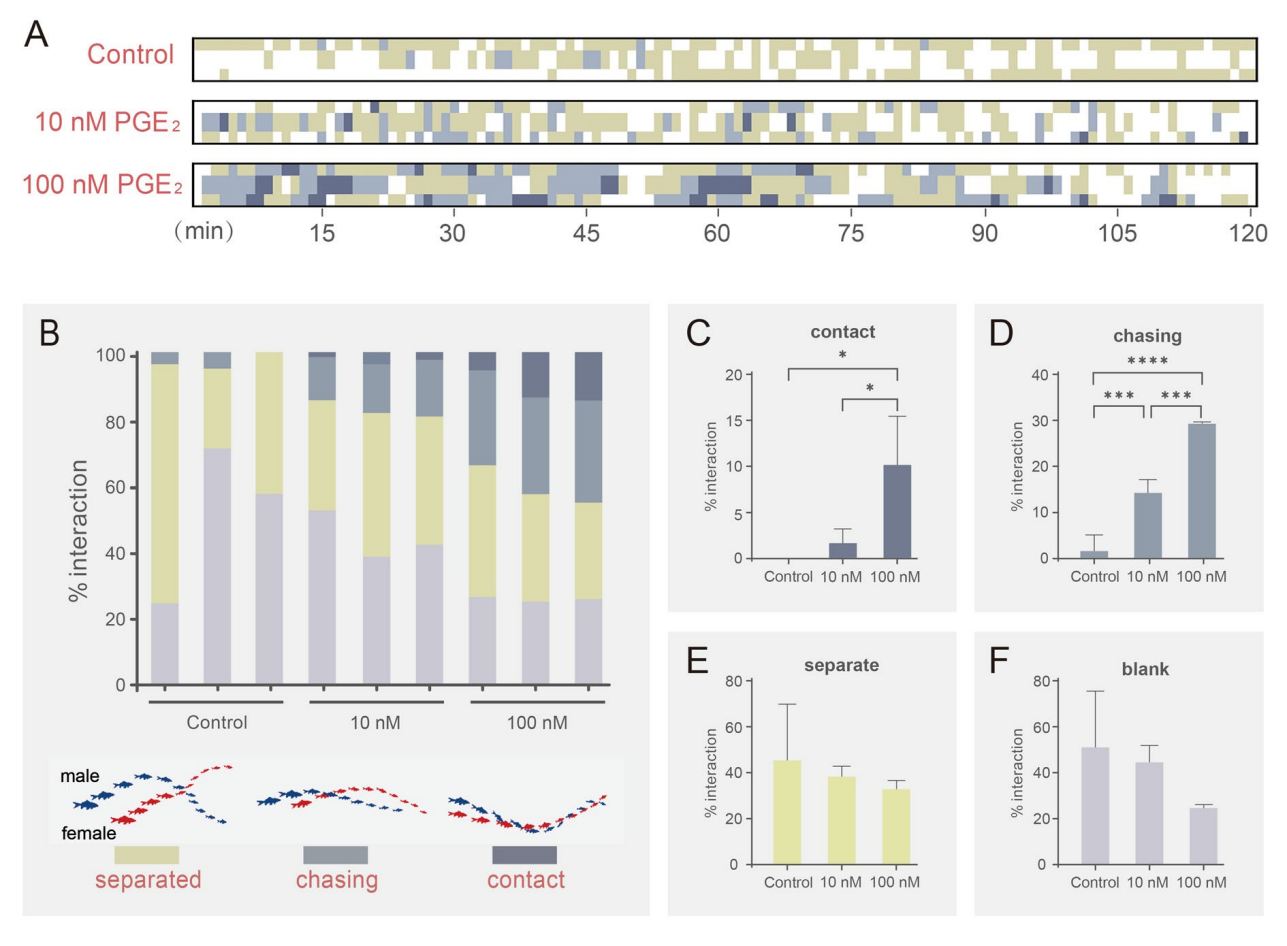
Behavior pattern analysis under PGE_2_ stimulation (*n* = 3). **A** Mating behavior heatmap of black rockfish. *X*-axis indicates the time after stimulation. *Y*-axis indicates PGE_2_ concentration (0, 10 nmol/L, 100 nmol/L). **B** Behavior pattern percentage of each treatment group. *X*-axis indicates nine experiments from three treatment groups. *Y*-axis indicates the percentage of each specific interaction. Blanks indicate no interaction during the experiment. **C**–**F** Statistical analysis of each specific interaction including contact, chasing, separate, and no interaction as blank. * represents *P* < 0.05; *** represents *P* < 0.001; **** represents *P* < 0.0001

To test the function of PGE_2_ in mating, we first observed the behavioral responses of adult male and female black rockfish under different concentrations of PGE_2_. The results showed that mating behavior, including chasing and contact, was elicited by 10 nmol/L PGE_2_ within 120 min. Furthermore, more intense behavior between males and females was observed when the concentration of PGE_2_ was increased to 100 nmol/L (Fig. 2A, B). Statistical analysis revealed that the percentage of contact interactions at 100 nmol/L PGE_2_ showed a significant difference (*P* < 0.05) compared with the 10 nmol/L or control groups (Fig. 2C). In addition, the percentage of contact interactions at 100 nmol/L PGE_2_ was significantly higher than in the 10 nmol/L (*P* < 0.001) or control groups (*P* < 0.0001) (Fig. 2D). However, no significant difference was observed in the percentage of separation and no interaction (blank) (Fig. 2E, F).

### PGE_2_ receptor identification in black rockfish mating behavior

**Fig. 3:**
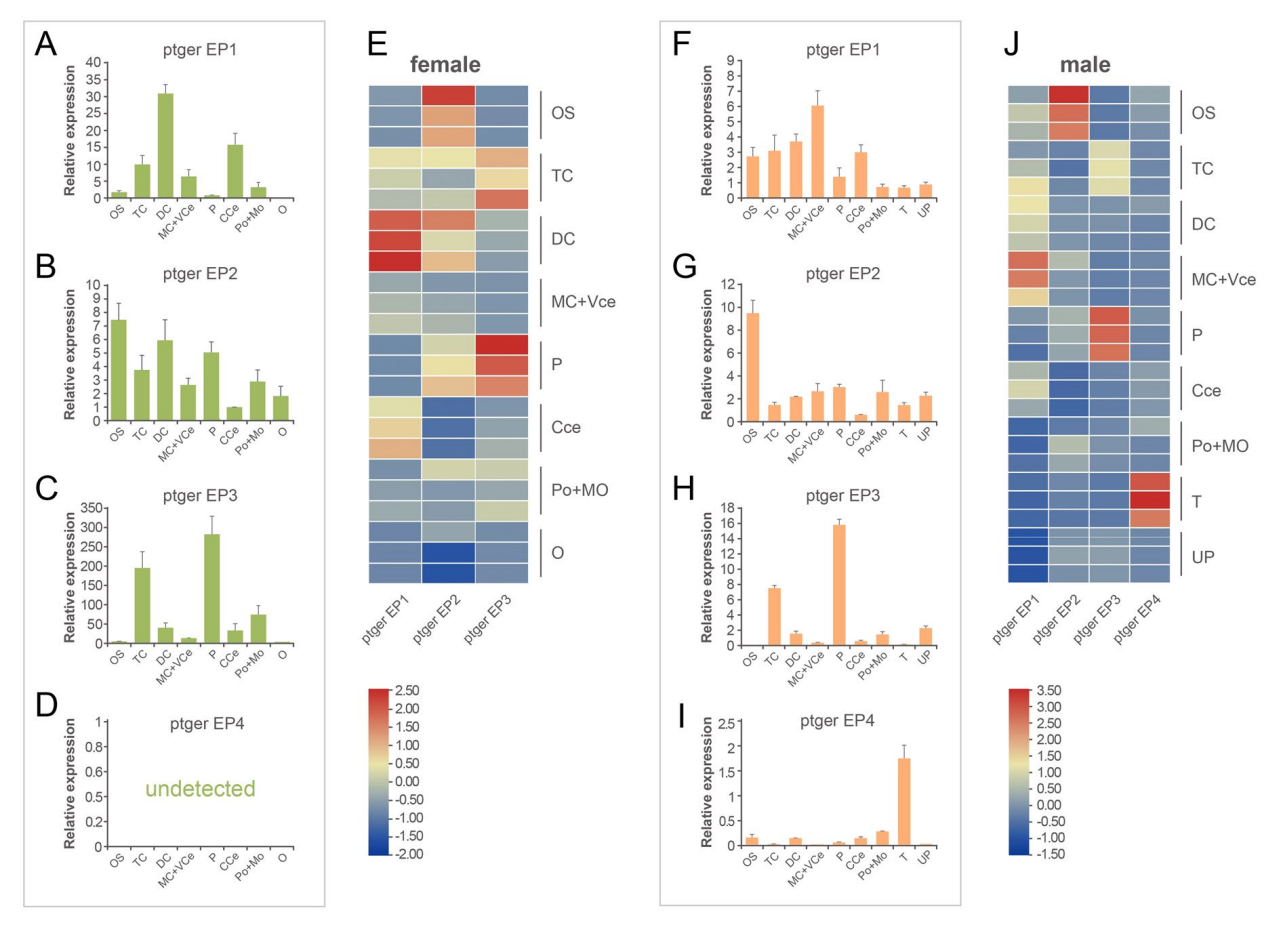
Expression level of *ptgers* in black rockfish peripheral olfactory system and central nervous system (*n* = 3). **A**–**D** Expression patterns of *ep1*, *ep2*, *ep3*, and *ep4* in females. **F**–**I** Expression patterns of *ep1*, *ep2*, *ep3*, and *ep4* in males. **E**, **J** Heatmap of four *ptger* expression patterns in females and males, respectively. *CCe* corpus cerebelli, *DC* diencephalon, *MC* mesencephalon, *MO* medulla oblongata, *OS* olfactory sac, *O* ovary, *P* pituitary, *Po* pons, *T* testis, *TC* telencephalon, *UP* urogenital papillae, *VCe* valvula cerebelli

Since PGE_2_ elicited a series of mating behaviors, subsequent studies focused on the functional receptors of PGE_2_. By RNA-seq and genomic database data mining, four PGE_2_ receptors, namely ptger EP1 (PGE_2_ receptor 1 subtype), EP2, EP3, and EP4, were identified. The expression levels of these four ptgers were tested by qPCR in the male and female peripheral olfactory system, central nervous system, and reproductive system in the mating season. In males, EP1 receptor mRNA was mainly detected in the DC region, followed by the CCe. The EP2 receptor was evenly distributed in different brain regions, while the highest expression level was in the OS. EP3 was mainly detected in P and TC. However, no EP4 signal was detected during mating in males (Fig. 3A–D). Meanwhile, EP1 mRNA was mainly detected in the MC + VCe region in the male brain. EP2 was significantly expressed in the OS compared with other parts of the brain and reproductive system. Similar to females, EP3 in males was also mainly expressed in P and TC. However, unlike females, EP4 was detected only in male testes (Fig. 3F–I). A heatmap demonstrated the expression patters of the four different *ptgers* in both sexes (Fig. 3E, J), which indicated that the potentially functional receptor in mating behavior was EP2.

### Localization of neuro cells activated by PGE_2_ in the brain and olfactory sac

**Fig. 4:**
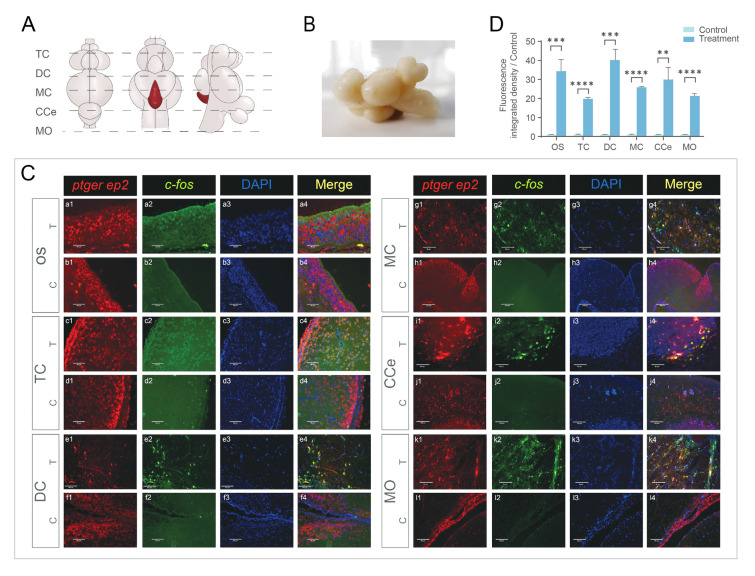
Dual-fluorescence in situ hybridization (DISH) colocalizationof *c-fos* (green, stained with Alexa Fluor 488) and *ep2* (red, stained with Alexa Fluor 594) in male black rockfish (*n* = 5). **A** Top view, bottom view, and lateral view of male black rockfish and the sketch map showing slice positions. **B** Image of male black rockfish brain in lateral view. **C** DISH staining in peripheral olfactory system and central nervous system in treatment group (T, 0.1 mg/mL PGE_2_ incubation) and control group (C). Cell nucleus was stained with DAPI (blue). Scale bar (a1–a4) = 30 μm. Scale bar (h1–h4) = 250 μm. Scale bar (j1–j4) = 130 μm. Scale bar (b1–b4, c1–c4, d1–d4, e1–e4, f1–f4, g1–g4, i1–i4, k1–k4, l1–l4) = 60 μm. **D** Fluorescence-integrated density of *c-fos* signal in DISH under PGE_2_ treatment (0.1 mg/mL) and control. Values of control group were set to 1. Bars represent mean values ± SEM. ** represents statistically significant difference (*P* < 0.01); *** represents highly statistically significant difference (*P* < 0.001); **** represents extremely highly statistically significant difference *P* < 0.0001. *CCe* corpus cerebelli, *DC* diencephalon, *MO* medulla oblongata, *MC* mesencephalon, *OS* olfactory sac, *TC* telencephalon

**Fig. 5:**
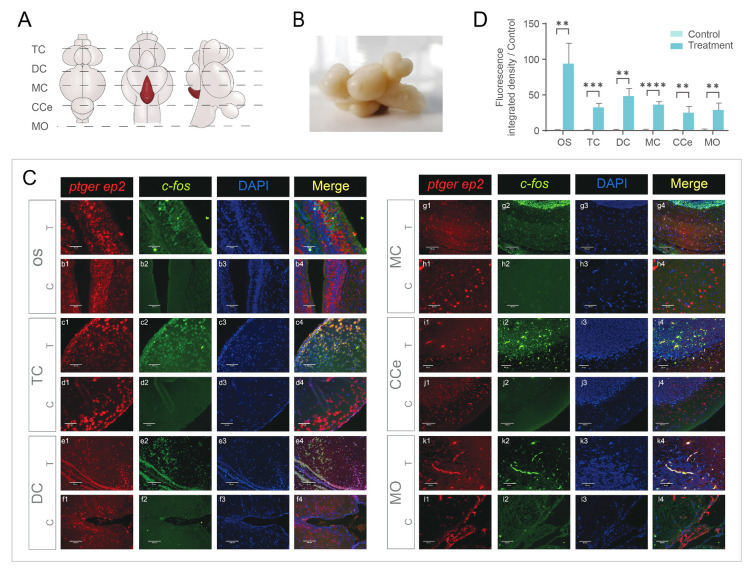
Dual-fluorescence in situ hybridization (DISH) colocalization of *c-fos* (green, stained with Alexa Fluor 488) and *ep2* (red, stained with Alexa Fluor 594) in female black rockfish (*n* = 5). **A** Top view, bottom view, and lateral view of female black rockfish and the sketch map showing slice positions. **B** Image of male black rockfish brain in lateral view. **C** DISH staining in peripheral olfactory system and central nervous system in treatment group (T, 0.1 mg/mL PGE_2_ incubation) and control group (C). Cell nucleus stained with DAPI (blue). Scale bar (a1–a4) = 30 μm. Scale bar (f1–f4, g1–g4, j1–j4) = 130 μm. Scale bar (b1–b4, c1–c4, d1–d4, e1–e4, h1–h4, i1–i4, k1–k4, l1–l4) = 60 μm. **D** Fluorescence-integrated density of *c-fos* signal in DISH under PGE_2_ treatment (0.1 mg/mL) and control. Values of control group were set to 1. Bars represent mean values ± SEM. ** represents statistically significant difference (*P* < 0.01); *** represents highly statistically significant difference (*P* < 0.001); **** represents extremely highly statistically significant difference *P* < 0.0001. *CCe* corpus cerebelli, *DC* diencephalon, *MO* medulla oblongata, *MC* mesencephalon, *OS* olfactory sac, *TC* telencephalon

The main functional olfactory PGE_2_ receptor in black rockfish mating behavior was EP2, which was also expressed in different brain regions. Together, PGE_2_ acts not only in the peripheral olfactory system but also in the central nervous system (CNS). To test whether PGE_2_ could also activate neurons in the CNS, DISH was performed to colocalize *ep2* and *c-fos* mRNA, which is a proxy for recent neural activity. DISH results in both sexes showed that the *ep2*-positive signal was mainly distributed in the olfactory epithelium in areas covering the tip surface of olfactory lamellae, and a few positive signals were also observed on the ridges of lamellae. In contrast, *ep2* and *c-fos* colocalization signals were detected only on ciliated receptor cells in the olfactory epithelium (Figs. 4C, 5C). In the TC, colocalization signals were detected in the pallium of the lateral part of the dorsal telencephalon (Dl) in both males and females. According to the qPCR results, EP2 was highly expressed in the female DC region, and the DISH results also showed a colocalization signal in the female hypothalamus (Hy). Positive signals were detected in the posterior recess in both sexes. In the male mesencephalon (MC) area, positive signals were observed in neuronal cell bodies in the optic tectum (TeO). In females, positive signals were only detected in a few small neuronal cells in the torus semicircularis (TS). The CCe area had low levels of *ep2* expression, and only a few signals were detected in male Purkinje cells. In the medulla oblongata (MO), few small neuronal cells showed positive signals. Negative control by sense probes results are provided in Supplementary Fig. S1 and Supplementary Fig. S2.

### Physiological and molecular changes after ICV administration of PGE_2_

The results of the present study implied that PGE_2_ can elicit mating behavior in black rockfish via the peripheral olfactory system. Furthermore, EP2 subtype receptors were expressed in different brain regions. Therefore, ICV was performed to analyze the effect of PGE_2_ on hormones and reproduction-related genes.

**Fig. 6:**
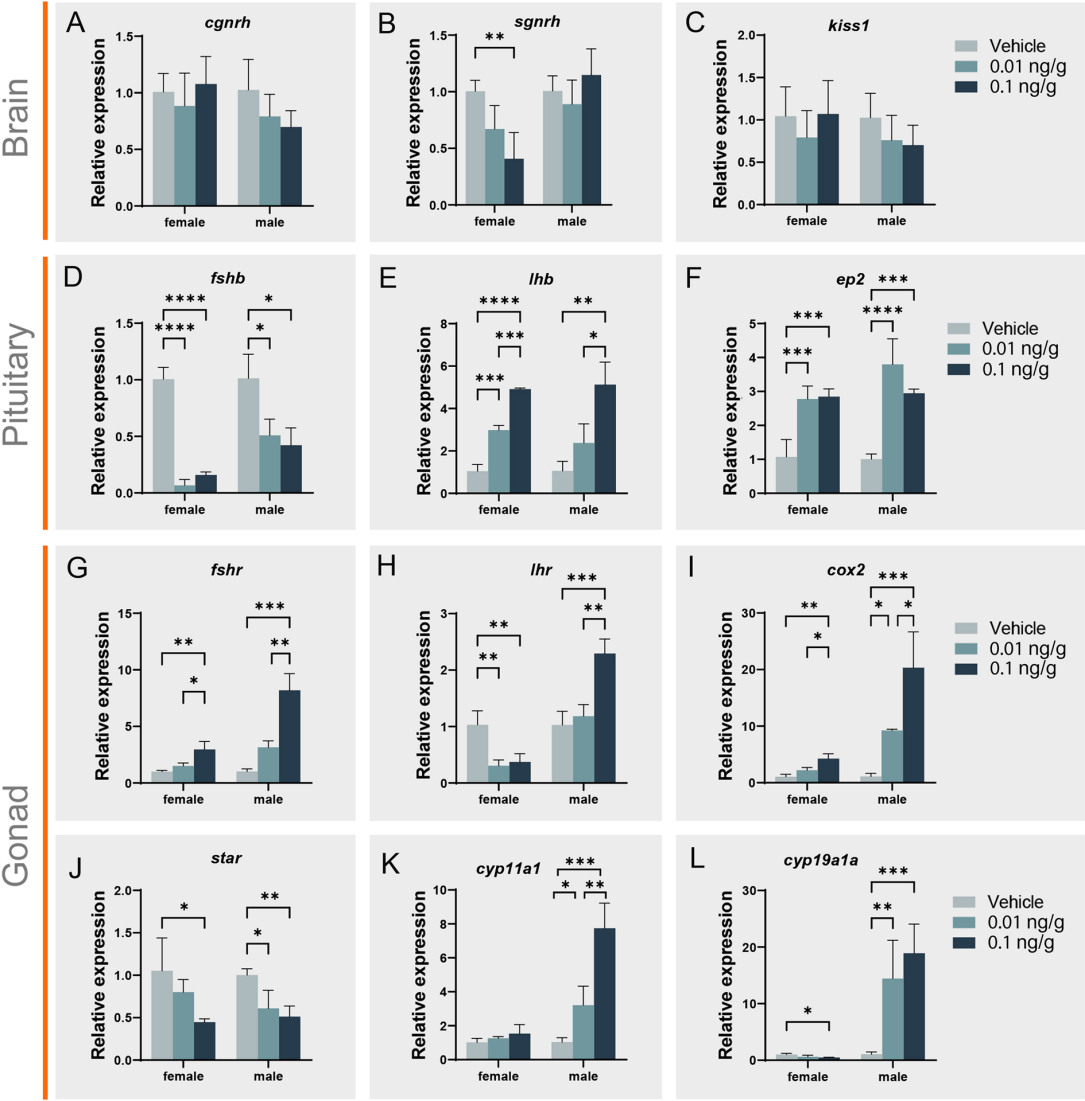
qPCR analysis the reproductive related genes expression pattern after ICV administration of PGE_2_ (0.01 ng/g, 0.1 ng/g) and control group (*n* = 3). **A**–**C** Expression pattern of genes in brain (*cgnrh*, *sgnrh* and *kiss1*). **D**–**F** Expression pattern of genes in pituitary (*fshb*, *lhb* and *ep2*). **G**–**L** Expression pattern of genes in gonad (*fshr*, *lhr*, *cox2*, *star*, *cyp11a1*, and *cyp19a1a*). The *X*-axis indicates injection with different concentrations of PGE_2_ in both sexes. The *Y*-axis indicates the relative expression normalized by 18S RNA. * represents statistic difference (*P* < 0.05); ** represents statistically significant difference (*P* < 0.01); *** represents highly statistically significant difference (*P* < 0.001); **** represents extremely highly statistically significant difference *P* < 0.0001

The qPCR results revealed that the mRNA levels of genes related to reproduction in the brain, including *kiss1*, *cgnrh*, and *sgnrh*, were not significantly different from each other (Fig. 6A–C). Gonadotropin (GtH) is a crucial factor in reproduction. In the present study, *fshb* and *lhb* showed various expression differences following PGE_2_ injection. The *fshb* mRNA level was significantly downregulated in females (*P* < 0.0001) and males (*P* < 0.05) at both PGE_2_ concentrations (Fig. 6D). In contrast, *lhb* mRNA was significantly increased in females (*P* < 0.001) and males (*P* < 0.05, Fig. 6E). Interestingly, the *ep2* level was significantly (*P* < 0.001) upregulated in the pituitary both in males and females (Fig. 6F). In gonads, the GtH receptor also presented various expression patterns. *Fshr* in both sexes and *lhr* in males were upregulated significantly in the two injection groups (*P* < 0.05). However, *lhr* in females was downregulated significantly (*P* < 0.01, Fig. 6G, H). COX-2, a key synthetase of PGE_2_, was upregulated in both sexes. In particular, the *cox2* level was upregulated only in the 0.1 ng/g group compared with the control (*P* < 0.01) and 0.01 ng/g groups (*P* < 0.01), whereas in males, levels were significantly higher (*P* < 0.05, *P* < 0.001) in both injection groups than in the controls (Fig. 6I). For a series of steroidogenesis-related enzymes, only *star* was significantly downregulated in both sexes (*P* < 0.05, Fig. 6J). Significant upregulation of *cyp11a1* was detected only in males (*P* < 0.001, Fig. 6K). *Cyp19a1a* was significantly upregulated in males (*P* < 0.001) and downregulated in females (*P* < 0.05, Fig. 6L).

**Fig. 7:**
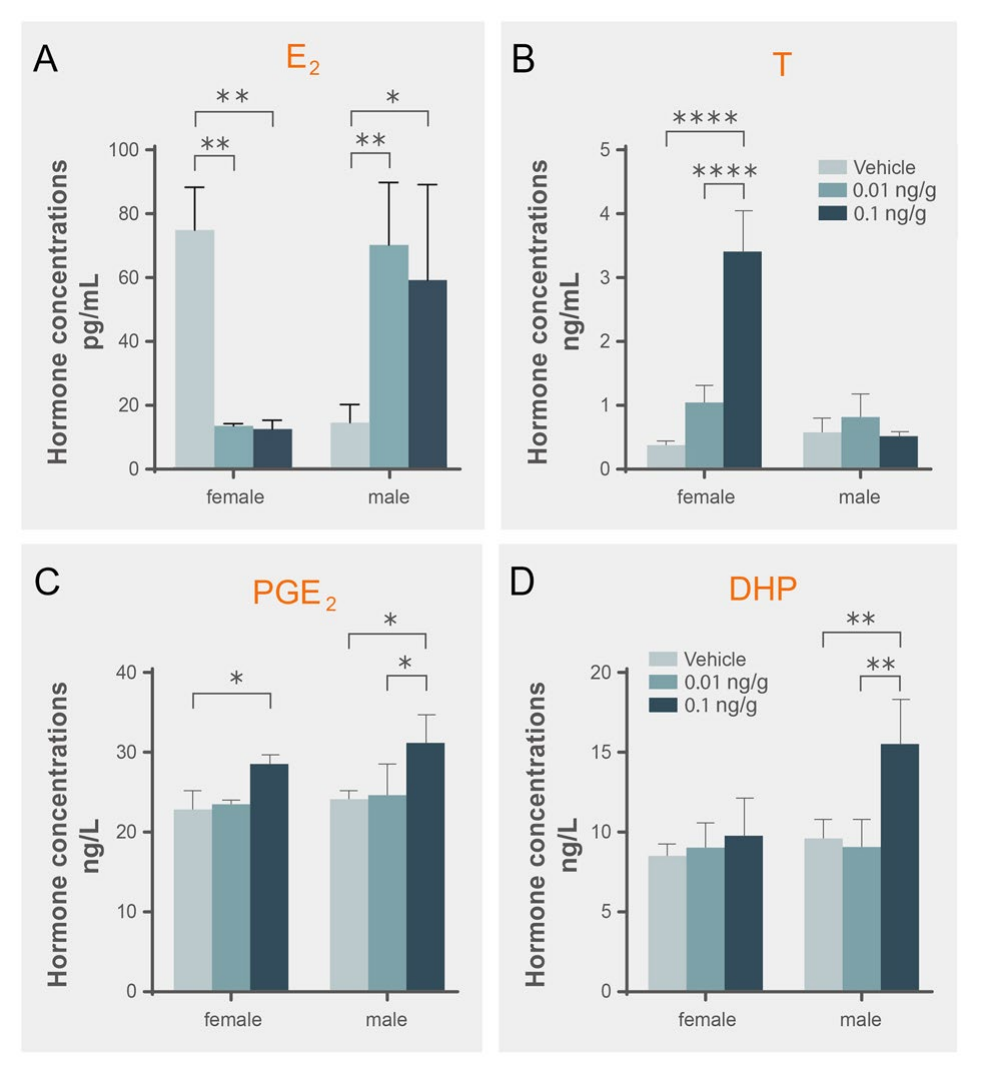
Hormone concentration after administration of PGE_2_ by ICV (*n* = 3). The *X*-axis indicates injection with different concentrations of PGE_2_ in both sexes. The *Y*-axis indicates the hormone concentrations of E_2_ (**A**), T (**B**), PGE_2_ (**C**), and DHP (**D**), respectively. * represents statistic difference (*P* < 0.05); ** represents statistically significant difference (*P* < 0.01); **** represents extremely highly statistically significant difference *P* < 0.0001

RIA results of ICV showed that the E_2_ concentration in females was significantly (*P* < 0.01) reduced from approximately 75 pg/mL (control group) to 13 pg/mL (0.01 ng/g or 0.1 ng/g injection group) (Fig. 7A). In contrast, in males, the serum E_2_ level was significantly higher than in the control group (14.56 pg/mL), and the E_2_ level was upregulated to 70.20 pg/mL after 0.01 ng/g injection (*P* < 0.01) and to 59.21 pg/mL after 0.1 ng/g injection (*P* < 0.05) (Fig. 7A). As the intermediate product of E_2_, the T concentration in females showed the opposite trend. The T level increased to 1.067 ng/mL and 3.425 ng/mL in the 0.01 ng/g and 0.1 ng/g injection groups, respectively, compared with the control group (0.397 ng/mL, *P* < 0.0001, Fig. 7B). However, no significant difference in T level change was observed in males (Fig. 7B). PGE_2_ levels were significantly increased in the 0.1 ng/g injection group in both males and females (*P* < 0.05, Fig. 7C). The DHP concentration was significantly induced (15.5 ng/L) in the 0.1 ng/g injection group in males compared with the control group and the 0.01 ng/g injection group (*P* < 0.01, Fig. 7D).

## Discussion

Fecundity is one of the most important factors for aquaculture fish species. As an ovoviviparous teleost, fecundity in black rockfish is reduced in comparison with oviparous fish taxa due to its special reproductive strategy (Haldorson and Love 1991). In the present study, the absolute brood amount in female black rockfish was positively correlated with body weight and body length, and individuals weighing over 1000 g had more than 100,000 fertilized eggs. It is suggested that in aquaculture purposes, maternal fish over 1000 g would be fit for reproduction. However, 36.67% of the population investigated here exhibited incomplete fertilization, which is consistent with that in a cage-cultured population of black rockfish in the Yellow Sea near Dalian in Liaoning Province (Luo et al. 2021). Therefore, incomplete fertilization has become one of the limiting factors for artificial reproduction in black rockfish.

Multiple paternity has been confirmed in previous studies on black rockfish (Leslie and Vrijenhoek 1977; Yoshida 2001). In a study on black rockfish in the Yellow Sea near Rushan and Penglai in Shandong Province, multiple paternity was observed from ten wild females (90.9%) with 2.45 sires on average. In an aquaculture population, 11 females were observed with multiple paternity (91.7%) with 3.08 sires on average (Gao et al. 2018). In a cage-cultured population of black rockfish in the Yellow Sea near Dalian in Liaoning Province, eight out of nine individuals were detected with multiple paternity, with an average sire number of 3.56. Interestingly, one of the eight individuals was incompletely fertilized (Luo et al. 2021). In the present study of black rockfish from an aquaculture farm in Rushan, Shandong Province, all ten individuals examined exhibited multiple paternity, with an average sire number of 5.20. In addition, higher sire numbers were found in the complete fertilization group, while the sire number in the incomplete fertilization group was 3.80. This suggests that a high mating success rate might be responsible for higher sire numbers and pregnancy rates.

Previous studies have indicated that PGs are functional in mating behavior alternation. In goldfish, PGF_2α_, which is crucial for ovulation, is released into water as postovulatory pheromones from ovulated females to stimulate males to perform their sexual behavior (Munakata and Kobayashi 2010). Similar sexual behavior patterns were also observed in female *Astatotilapia burtoni* intraperitoneally injected with PGF_2α_. In *A. burtoni*, brain cells can transduce PGF_2α_ signals to mate (Juntti et al. 2016). In zebrafish, PGF_2α_ has been shown to activate two olfactory receptors as pheromones to induce male reproductive behaviors (Yabuki et al. 2016). Another report in zebrafish also indicated that E_2_ exposure can alter mating behavior (Pradhan and Olsson 2015). Moreover, a study on the Chinese black sleeper (*Bostrychus sinensis*) showed that PGE_2_-releasing tubes attract more males and females with higher spawning rates than the control group (Hong et al. 2006). The EOG response to PGE_2_ in the mature *B. sinensis* olfactory system is greater than that in immature fish (Zhang et al. 2019). Furthermore, in guppies, which like black rockfish are ovoviviparous teleosts, 30 nmol/L PGE_2_ can trigger courtship between female and males compared with the control group (unpublished data). In the present study, we tested the function of PGs in eliciting the mating behavior of black rockfish. The results revealed that PGE_2_ in the water can promote interactions between females and males. Furthermore, PGE_2_ was shown to activate epithelial receptor cells in the peripheral olfactory system and neurons in the central nervous system, and EP_2_ subtype was the main functional receptor during mating. All these findings indicate the potential function of PGE_2_ as a sex pheromone in teleosts.

PGs act not only as pheromones, but also as hormones that function in the HPG axis and central nervous system. PGF_2α_ injection in a female *Cichlasoma bimaculatum* at any stage in the spawning cycle or parental phase induces rapid substrate cleaning and spawning behavior without egg release (Cole and Stacey 1984). In a study on *Astatotilapia burtoni*, the PGF_2α_ receptor Ptgfr and *c-fos* mRNA were located in special cells in the POA after being allowed to spawn naturally (Juntti et al. 2016). These results indicate that PGF_2α_ conveys to the brain of females information on the presence of ovulated oocytes in the ovary and their readiness to be oviposited (Munakata and Kobayashi 2010). In the present study, colocalization signals for *ep2* and *c-fos*, a proxy for recent neural activity, were observed in different brain regions after PGE_2_ stimulation. These findings suggest that PGE_2_ can not only function as a pheromone to attract males, but also act as an endogenous hormone to regulate the neuroendocrine system in both males and females.

To test the direct effect of PGE_2_ on the neuroendocrine system, its administration by ICV was performed on male and female black rockfish during the mating season. Previous studies have mainly focused on which PG syntheses are regulated by GtH, especially LH (Piotrowska-Tomala et al. 2020; Tang et al. 2017). Less is known about how PGs participate in GtH expression and release. Injection of PGE_2_ and PGF_2α_ into the third ventricle of goldfish results in significant decreases in serum GtH levels (Peter and Billard 1976). PGE_2_ injected into the third ventricle of rats increases LH dramatically and FSH slightly (Harms et al. 1973). These findings suggest that PGE_2_ may directly stimulate GtH levels. Similar to these reports, administration of PGE_2_ by ICV in black rockfish resulted in significant upregulation of *lhb* and downregulation of *fshb*. Generally, neuropeptides including GnRH (sGnRH and cGnRH) and kisspeptin are accepted as GtH regulators. Previous studies revealed that PGE_2_ from hypothalamic astrocytes and tanycytes can stimulate GnRH secretion as a gliotransmitter (Clasadonte et al. 2011). However, in the present study, *sgnrh*, *cgnrh* and *kiss1* showed no difference following the administration of PGE_2_ by ICV, except that the *sgnrh* level was decreased in female black rockfish. Moreover, *ep2* increased in the pituitary after ICV, which is consistent with the direct regulatory effect of PGE_2_ on GtH.

**Fig. 8:**
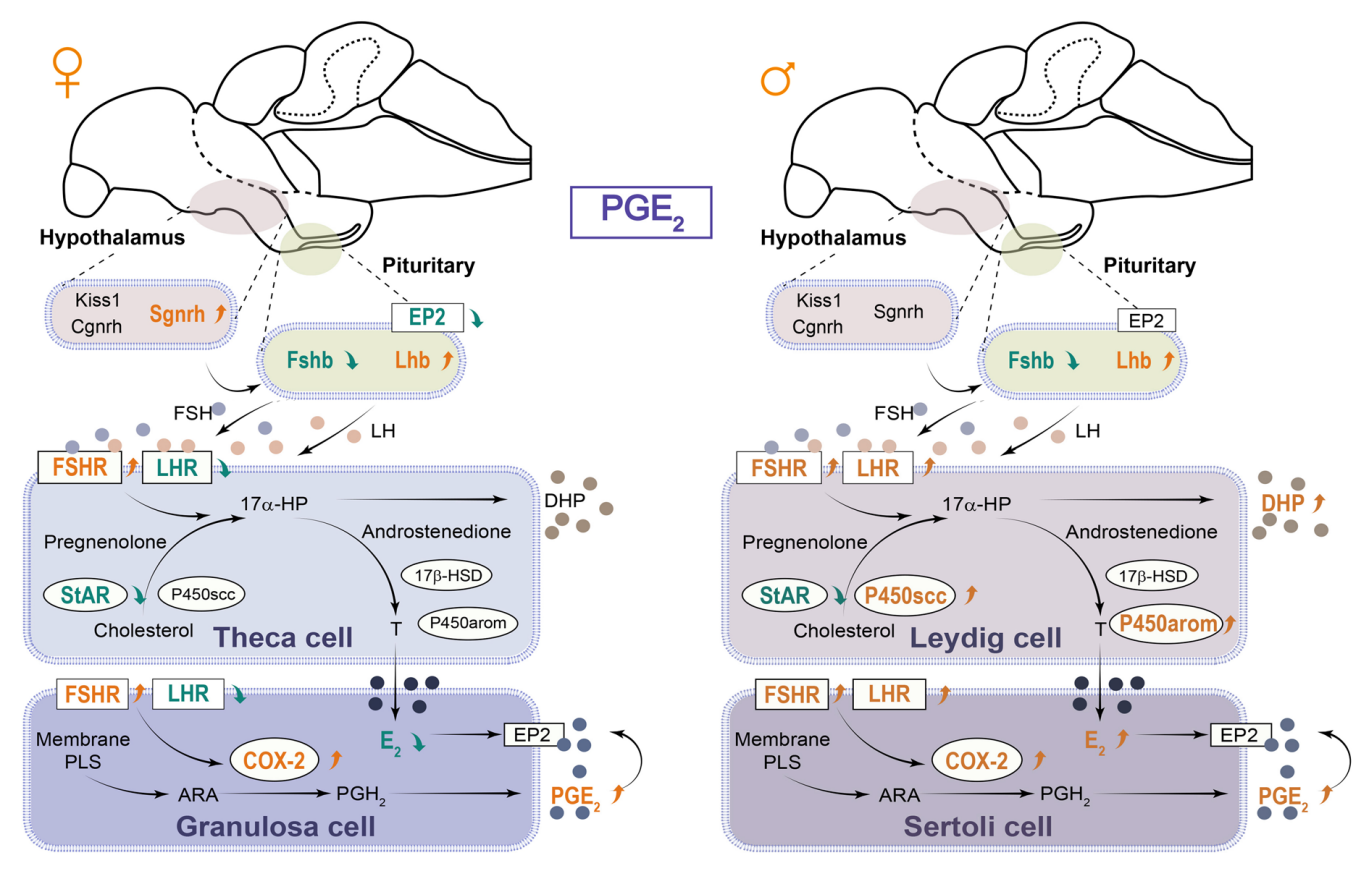
Regulatory mechanism of PGE_2_ administered by ICV in both sexes

Following the change in GtH, steroidogenesis in the gonads also showed differences. The *challenge hypothesis* suggests that androgens and reproductive aggression in adult male animals are closely associated (Wingfield et al. 1990). Furthermore, androgen levels and time spent on courtship behavior are related in male blenniid fish (*Rhabdoblennius nitidus*), and cyproterone acetate, an antiandrogen, can shorten the time on courtship (Matsumoto et al. 2012). A study on female zebrafish showed an increase in sexuality levels and characteristic swimming patterns for mating after 30 days of treatment with a high level of T and separation from males (Liu et al. 2021). Moreover, alterations in reproductive behavior were observed when male zebrafish were exposed to E_2_ and female zebrafish were exposed to 11-KT (Pradhan and Olsson 2015), which implied the complexity of sex steroids on reproductive behavior patterns. Zebrafish exposed to EE2 (17α-ethinyl estradiol, a synthetic estrogen) exhibit sex reversal from male to female, and the males that do not undergo sex reversal show either unaltered male sexual behavior or reduced sexual behavior (Colman et al. 2009; Larsen et al. 2008; Nash et al. 2004). Mature male goldfish exposed to E_2_ exhibit severely affected reproductive behavior and physiology (Bjerselius et al. 2001). Male guppies in EE2 spend more time performing “sigmoid” displays (a term of courtship display in guppies toward the visual cues of females) (Saaristo et al. 2019). In the present study, the T level of female black rockfish significantly increased after administration of PGE_2_ by ICV, which is a consequence of the decrease in *cyp19a1a* and E_2_ levels, and may be responsible for behavior alternation in females. In addition, as ovoviviparous teleosts, mating and ovulation in black rockfish are dissociated. It is further indicated that steroids may also participate in sexual behavior (Stacey 1981). In males, the E_2_ level was significantly increased. On the one hand, E_2_ may act on the brain and affect mating behavior, which is consistent with the results from brain transcriptomic data after EE2 exposure in guppies (Saaristo et al. 2021). On the other hand, E_2_ can regulate ptger expression levels (Blesson et al. 2012), and PGE_2_ may have an influence on sperm mobility (Carlson et al. 2022; Kennedy et al. 2003). Further studies are required in order to elucidate this mechanism in teleosts. It is noteworthy that DHP levels also increase in males, which can not only induce spermiation, but also modulate prostaglandin receptor mRNA levels (Juntti et al. 2016; Schulz et al. 2010). Taken together, PGE_2_ in the water could trigger the peripheral olfactory system and central nervous system. Moreover, PGE_2_ functions in the brain to activate steroidogenesis by regulating GtH levels, leading to a series of steroid differences (Fig. 8) and potentially increasing the probability of mating success in black rockfish.

## Conclusions

In summary, our results revealed that PGE_2_: (1) is a functional molecule that activates black rockfish mating behavior; (2) activates the peripheral olfactory system and CNS by binding to EP2 receptor; (3) activates *lhb* levels and steroidogenesis following ICV administration; (4) activates mating behavior in black rockfish via both the hormone and the pheromone pathways, leading to variation in sex steroid levels and activation of reproductive behaviors.

### Supplementary Information

Below is the link to the electronic supplementary material.

Supplementary file1 (PDF 570 KB)

## Data Availability

The datasets generated during and/or analysed during the current study are included in this published article (and its supplementary file).
