# Supplementary material for: Mating behaviors in ovoviviparous black rockfish (Sebastes schlegelii): molecular function of prostaglandin E2 as both a hormone and pheromone
Source: Mar Life Sci Technol. 2024 Jan 19;6(1):15–30. doi: 10.1007/s42995-023-00214-w (PMC10902245; doi:10.1007/s42995-023-00214-w)

Mating behaviors in ovoviviparous black rockfish (*Sebastes schlegelii*): molecular function of prostaglandin E2 as both a hormone and pheromone

Likang Lyu[1], Yijia Yao[1], Songyang Xie[1], Xiaojie Wang[1], Haishen Wen[1], Yun Li[1], Jianshuang Li[1], Chenpeng Zuo[1], Shaojing Yan[1], Jingyi Dong[2], Xin Qi[1*]

[1] Key Laboratory of Mariculture (Ocean University of China), Ministry of Education, Ocean University of China, Qingdao 266003, China

[2] Institute of Evolution & Marine Biodiversity, Ocean University of China, Qingdao 266003, China

*Address all correspondence to:

Xin Qi, PhD

Key Laboratory of Mariculture, Ministry of Education, Ocean University of China

Qingdao 266033, China.

Tel: +86-532-82031792,

Email: qx@ouc.edu.cn

Supplementary Table 1 Biological indexes of black rockfish during pregnancy.

	body weight (g)	body length (cm)	gonad weight (g)	absolute brood amount	fatness	pregnancy rate (%)
1	1050.2	38.21	276.56	101022.4342	1.882526	100
2	1200.4	39.4	265.79	89779.30964	1.962625	100
3	790.25	34.43	151.93	80333	1.936214	87.43
4	1600.4	41.91	380.48	184386.4615	2.17408	100
5	1200.1	39.22	175.75	110299.8731	1.989274	59.57027487
6	681.5	33.87	114.13	52419	1.753962	93.64
7	700.65	33.61	136.5	65119.26606	1.845422	100
8	920.7	35.78	205.53	97309	2.010005	100
9	950.2	36.57	148.46	76982	1.942854	87.99
10	850.1	35.12	89.79	55754	1.962486	37.81
11	1150.3	37.23	210.35	101938.8462	2.229113	100
12	1150.5	37.55	179.45	93051.05319	2.172985	91.47
13	950.5	35.17	176.53	85549.15385	2.184917	100
14	904.5	34.54	201.45	97625.76923	2.195036	100
15	1060.4	37.14	281.35	136346.5385	2.069875	100
16	1050	37.42	201.54	125143.8707	2.003909	82.46
17	950.3	36.96	181.33	77570.68972	1.882196	100
18	950.2	35.49	188.46	80620.8139	2.125676	100
19	915.3	34.43	157.77	81809.22074	2.242603	90.57
20	950.5	34.51	179.48	76779.28302	2.312689	100
21	845.2	34.52	133.67	57182.34211	2.054694	100
22	1030.5	36.04	184.45	114532.0381	2.201373	78.34
23	780.5	32.22	152.77	74034.69231	2.333438	100
24	1350.4	39.83	340.35	164938.8462	2.137133	100
25	1250.5	38.57	317.98	154098	2.179389	100
26	1350.5	39.37	377.46	182922.9231	2.213086	100
27	970.4	34.5	203.55	98643.46154	2.363162	100
28	830.3	36.76	96.34	49955.63368	1.671508	89.93
29	835.3	35.5	90.33	56089.34129	1.867056	80.78
30	1100.4	36.07	225.69	109372.8462	2.344834	100

Supplementary Fig. 1 *Ptger ep2* and *c-fos* sense probe results of DISH in female black rockfish olfactory sac and brain.

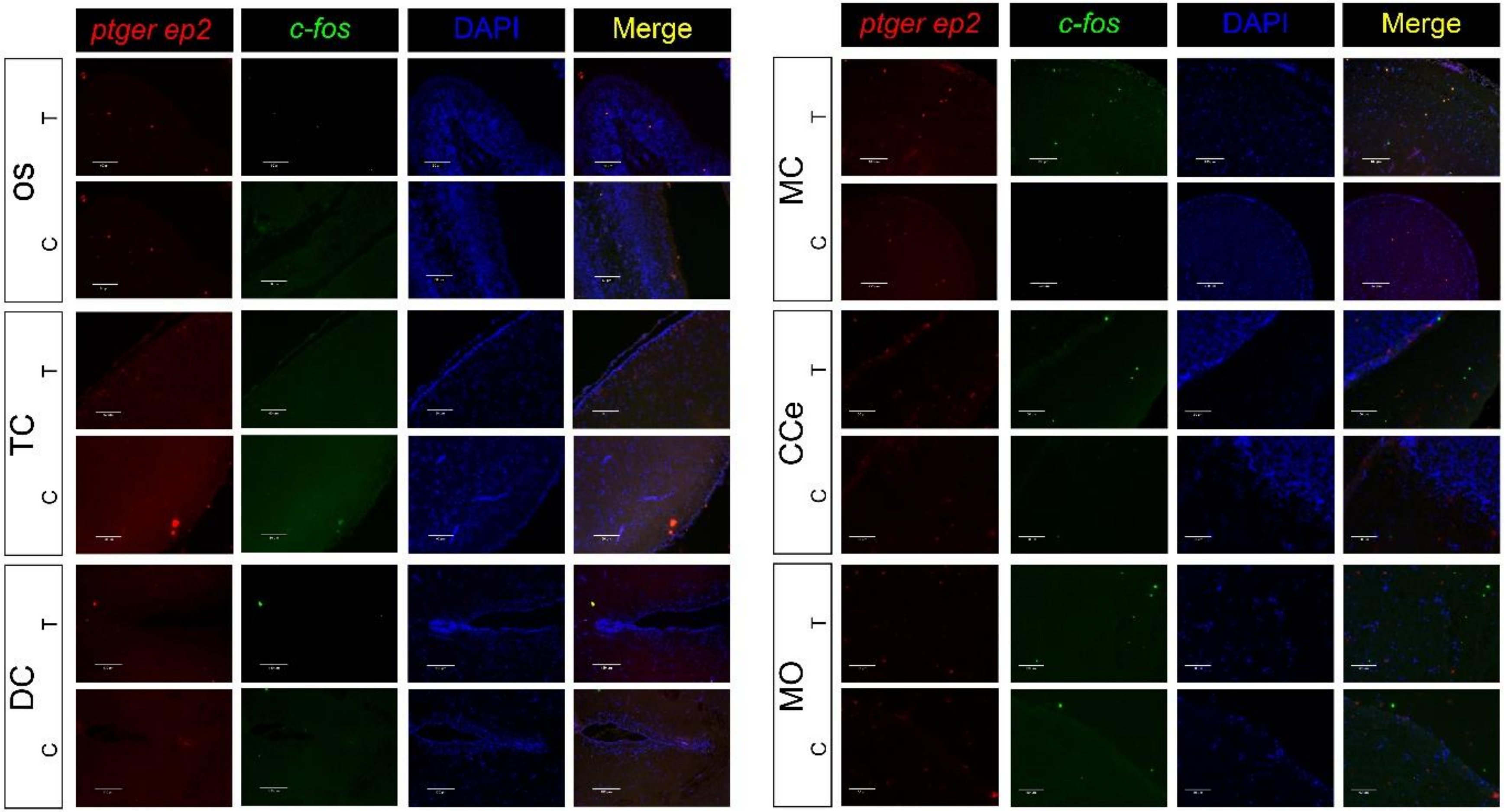

Supplementary Fig. 2 *Ptger ep2* and *c-fos* sense probe results of DISH in male black rockfish olfactory sac and brain.

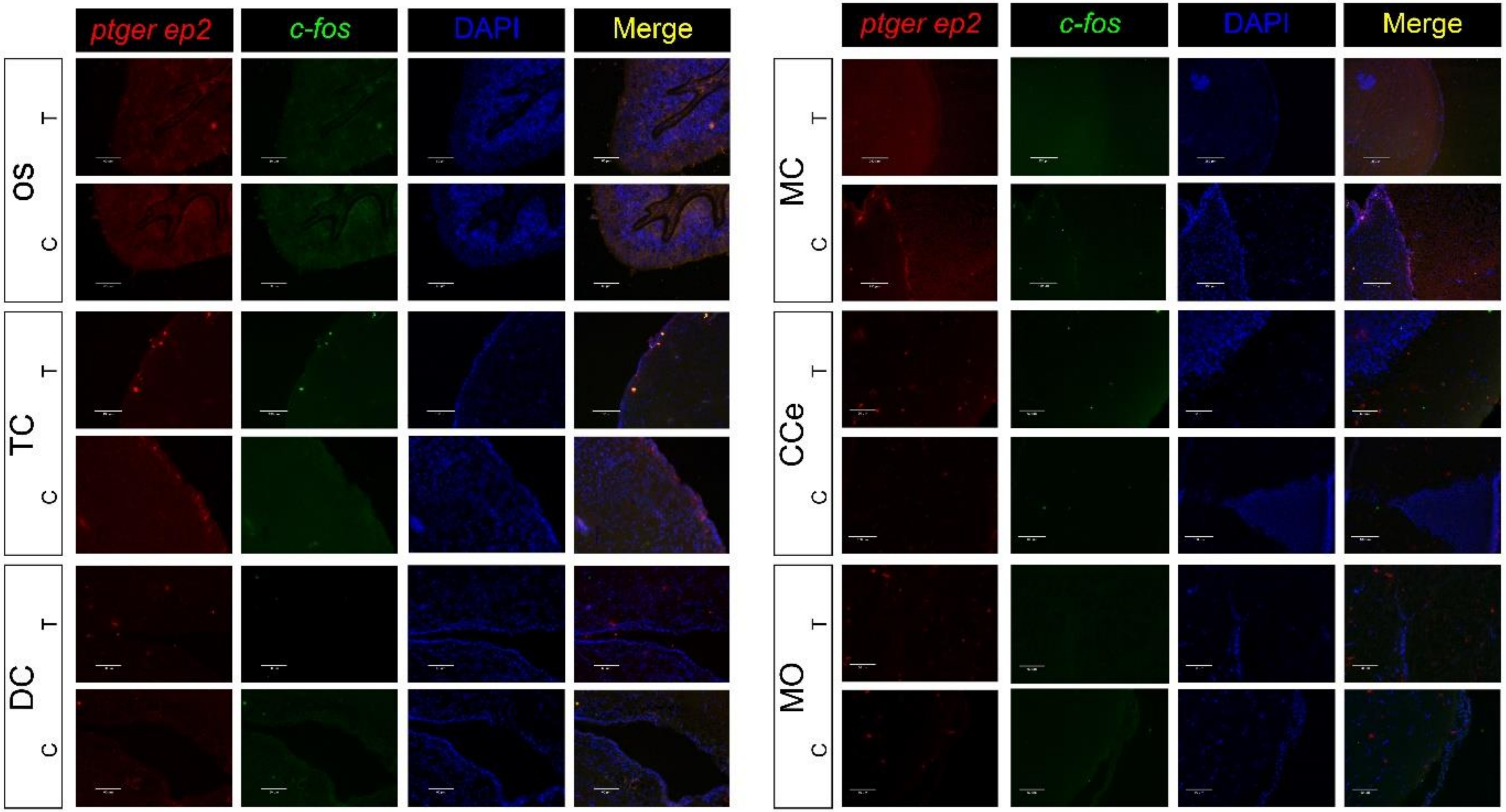

Supplement: Supplementary file 1 — Supplementary file1 (PDF 570 KB) [file 42995_2023_214_MOESM1_ESM.pdf]
